# Reciprocal regulation of endothelial–mesenchymal transition by MAPK7 and EZH2 in intimal hyperplasia and coronary artery disease

**DOI:** 10.1038/s41598-021-97127-4

**Published:** 2021-09-07

**Authors:** Byambasuren Vanchin, Marloes Sol, Rutger A. F. Gjaltema, Marja Brinker, Bianca Kiers, Alexandre C. Pereira, Martin C. Harmsen, Jan-Renier A. J. Moonen, Guido Krenning

**Affiliations:** 1 Laboratory for Cardiovascular Regenerative Medicine, Department of Pathology and Medical Biology, University Medical Center Groningen, University of Groningen, Hanzeplein 1 (EA11), 9713GZ Groningen, The Netherlands; 2 Department of Cardiology, School of Medicine, Mongolian National University of Medical Sciences, Jamyan St 3, Ulaanbaatar, 14210 Mongolia; 3 Laboratory of Genetics and Molecular Cardiology (LIM13), Heart Institute (InCor), University of São Paulo, Avenida Dr. Eneas C. Aguiar 44, São Paulo, SP 05403-000 Brazil; 4 Department of Pediatric Cardiology, Center for Congenital Heart Diseases, Beatrix Children’s Hospital, University Medical Center Groningen, University of Groningen, Hanzeplein 1 (CA40), 9713GZ Groningen, The Netherlands

**Keywords:** Vascular diseases, Pathogenesis, Epigenetics, Non-coding RNAs, Cell signalling, Mechanisms of disease

## Abstract

Endothelial–mesenchymal transition (EndMT) is a form of endothelial dysfunction wherein endothelial cells acquire a mesenchymal phenotype and lose endothelial functions, which contributes to the pathogenesis of intimal hyperplasia and atherosclerosis. The mitogen activated protein kinase 7 (MAPK7) inhibits EndMT and decreases the expression of the histone methyltransferase Enhancer-of-Zeste homologue 2 (EZH2), thereby maintaining endothelial quiescence. EZH2 is the catalytic subunit of the Polycomb Repressive Complex 2 that methylates lysine 27 on histone 3 (H3K27me3). It is elusive how the crosstalk between MAPK7 and EZH2 is regulated in the endothelium and if the balance between MAPK7 and EZH2 is disturbed in vascular disease. In human coronary artery disease, we assessed the expression levels of MAPK7 and EZH2 and found that with increasing intima/media thickness ratio, MAPK7 expression decreased, whereas EZH2 expression increased. In vitro, MAPK7 activation decreased EZH2 expression, whereas endothelial cells deficient of EZH2 had increased MAPK7 activity. MAPK7 activation results in increased expression of microRNA (miR)-101, a repressor of EZH2. This loss of EZH2 in turn results in the increased expression of the miR-200 family, culminating in decreased expression of the dual-specificity phosphatases 1 and 6 who may repress MAPK7 activity. Transfection of endothelial cells with miR-200 family members decreased the endothelial sensitivity to TGFβ1-induced EndMT. In endothelial cells there is reciprocity between MAPK7 signaling and EZH2 expression and disturbances in this reciprocal signaling associate with the induction of EndMT and severity of human coronary artery disease.

## Introduction

Neointimal hyperplasia is characterized by an increasing amount of fibroproliferative cells and extracellular matrix in the neointimal lesion, resulting in vascular lumen narrowing—characterized by an increasing intima/media thickness ratio (IMT) in histopathology—and eventually obstruction of the vessel. Endothelial cells play a pivotal role in the formation of neointimal lesions by the acquisition of a fibro-proliferative phenotype through endothelial-to-mesenchymal transition (EndMT)^1–5^. EndMT is characterized by a change from an endothelial phenotype into a phenotype comprising of mesenchymal-like properties, in which the expression of endothelial cells markers, such as eNOS, PECAM-1 and VE-cadherin is lost, and the expression of mesenchymal genes, including SM22α, αSMA and vimentin, is gained. Moreover, EndMT-derived fibroproliferative cells secrete extracellular matrix components, which might contribute to the buildup of the neointima^6^.

EndMT was originally identified during embryogenesis, where it plays a pivotal role in cardiac valve, septum and endocardial cushion formation^7^. In adults, EndMT contributes to fibroproliferative diseases, including atherosclerosis^1–5^, cerebral cavernous malformation^8^, pulmonary fibrosis^9^, kidney fibrosis^10^ and cardiac fibrosis^11^.

Uniform laminar shear stress (LSS) conveys atheroprotective effects to the endothelium, while endothelial cells exposed to disturbed or low oscillatory shear stress are prone to EndMT^12,13^. Uniform LSS activates the mitogen-activated protein kinase 7 (MAPK7)—also known as extracellular signal-related kinase 5 (Erk5) and big-mitogen kinase-1 (BMK-1)—which suppresses EndMT^5,14,15^. Concurrently, the loss of MAPK7 signaling facilitates EndMT^5,16^. Currently, it is elusive how MAPK7 activity is regulated in fibroproliferative disease.

The histone methyltransferase Enhancer of Zeste Homolog 2 (EZH2), which is the catalytic subunit of the Polycomb Repressive Complex 2, plays a pivotal role in endothelial dysfunction^17–19^. EZH2 is responsible for the trimethylation of lysine 27 on histone 3, which silences gene expression and is elevated in endothelial cells in atherosclerotic lesions^20^. Serendipically, we uncovered that uniform LSS reduces the expression of EZH2, whereas the RNAi-mediated repression of EZH2 reciprocally activates MAPK7 signaling in endothelial cells even in the absence of LSS^18^. Currently, it is elusive how the crosstalk between MAPK7 and EZH2 is regulated in the endothelium and whether the balance between MAPK7 and EZH2 is disturbed during intimal hyperplasia and coronary artery disease.

Here, we report that in the endothelium there is reciprocity between MAPK7 and EZH2 in the regulation of EndMT and in human coronary artery disease. In endothelial cells exposed to uniform LSS, the activation of MAPK7 increases the expression of microRNA (miR)-101, which in turn suppresses the translation of the EZH2 gene, resulting in reduced protein expression. Reciprocally, the reduced expression of EZH2 results in the decreased expression of the Dual Specificity Phosphatase (DUSP)-1 and DUSP-6—the phosphatases responsible for the inactivation of MAPK7^21^—which facilitates the activation of MAPK7. Disturbance in the reciprocity between MAPK7 and EZH2 result in the induction of EndMT and associate to the degree of human coronary artery disease.

## Results

### Reciprocity between MAPK7 and EZH2 in human coronary artery disease

**Figure 1:**
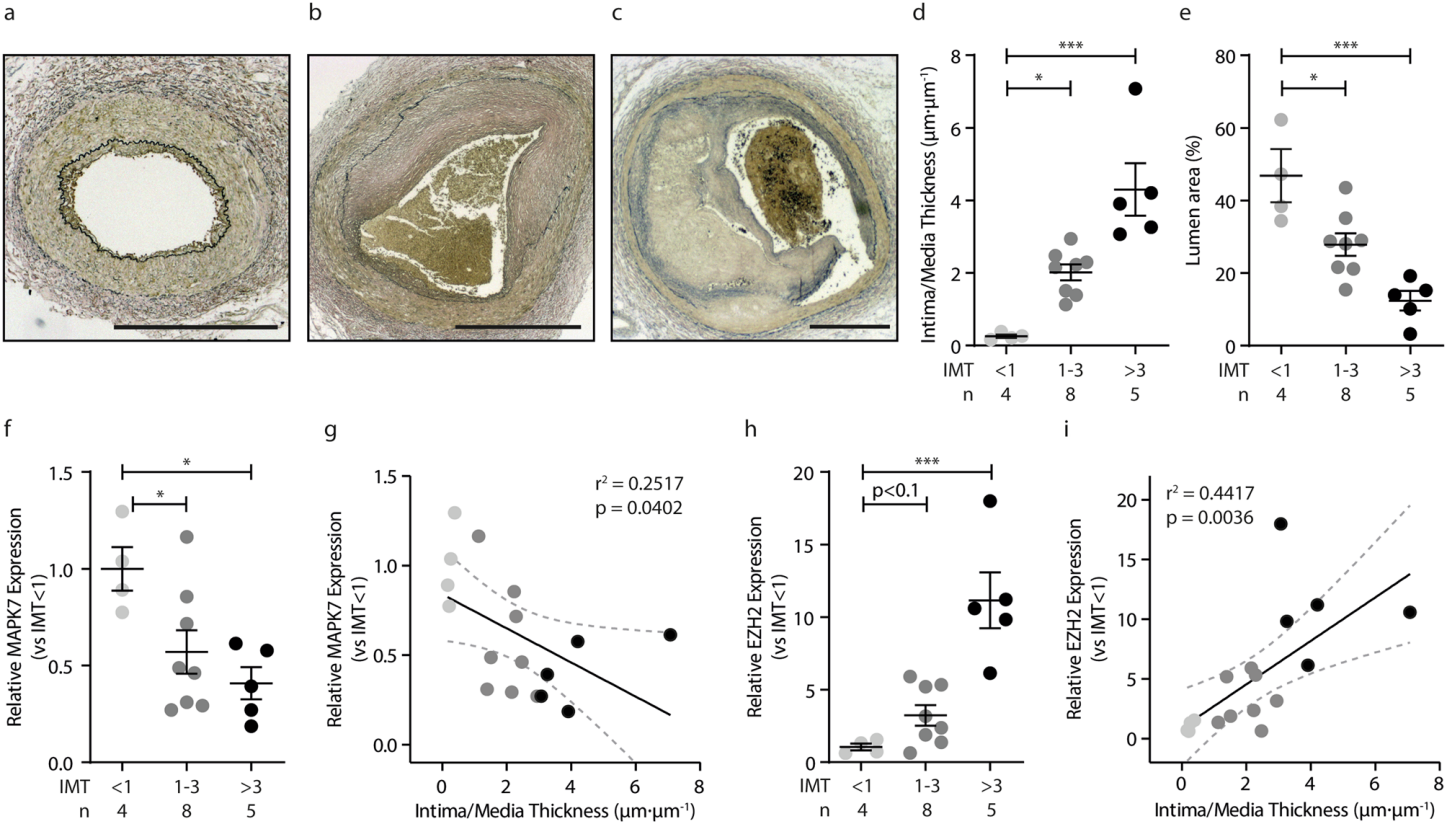
Reciprocity between MAPK7 and EZH2 in human coronary artery disease. **(a–c)** Representative pictures of Verhoeff-stained human coronary artery samples (n = 4–8) with increasing IMT, IMT < 1 **(a)** IMT 1–3 **(b)** and IMT > 3 **(c)**. Intima-media thickness was measured (µm_intima_/µm_media_) and samples stratified into three groups based on their intima-media thickness **(d)**. An increasing IMT coincides with a progressively decreasing lumen area of the coronary artery **(e)**, suggestive of progressive stenosis. *MAPK7* expression levels were determined by qPCR and normalized to IMT < 1 **(f)**. *MAPK7* decreases with increasing IMT **(g)**. *EZH2* expression levels were determined by qPCR and normalized to IMT < 1 **(h)**. *EZH2* expression increased with increasing IMT **(i)**. Data is expressed as mean ± S.D. of all individual observations. Statistical analysis was performed by ANOVA followed by Bonferroni post hoc tests. Correlations were performed using Pearson correlation. *p < 0.05, ***p < 0.001.

Human coronary artery samples were stratified into three groups based on their intima/media thickness ratio (IMT), ranging from IMT < 1 µm_intima_·µm_lumen_^−1^ to IMT > 3 µm_intima_·µm_lumen_^−1^; Fig. 1a–d). Mean age, and the hypertension, diabetes and smoking status did not differ between groups. Coronary artery disease is characterized by a progressively increasing intima-media thickness ratio (Fig. 1a–d), which coincides with the progressive narrowing of the coronary artery lumen (Fig. 1e). Coronary artery *MAPK7* expression decreases with an increasing coronary artery intima-media thickness ratio (r^2^ = 0.2517, p = 0.04; Fig. 1f,g). In contrast, *EZH2* expression is elevated in coronary artery disease (Fig. 1h,i) and an increasing coronary artery intima-media thickness ratio associates with increased *EZH2* expression (r^2^ = 0.4417, p = 0.004, Fig. 1i), suggesting reciprocity between MAPK7 and EZH2 in human coronary artery disease.

### Reciprocal signaling between MAPK7 and EZH2 in endothelial cells

**Figure 2:**
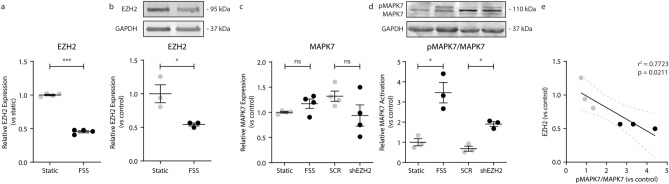
Reciprocal signaling between MAPK7 and EZH2 in endothelial cells. *EZH2* expression levels were determined by qPCR in HUVEC exposed to FSS (20 dyne/cm^2^) compared to static controls **(a)**. EZH2 protein levels were determined by western blot in HUVEC exposed to FSS and compared to static control **(b)**. *MAPK7* expression levels were determined by qPCR in HUVEC exposed to FSS, and HUVEC that are deficient in EZH2 (shEZH2) **(c)**. MAPK7 activation (pMAPK7) levels were determined by immunoblotting and normalized to total MAPK7 protein levels **(d)**. Protein expression of EZH2 and MAPK7 activation were associated in endothelial cells **(e)**. Data is expressed as mean ± S.D. of all individual observations. Gene and protein expression data were obtained from 4 and 3 independent experiments, respectively. Comparisons between 2 groups were performed by Student t-tests and data from multiple groups were analyzed by ANOVA followed by Bonferroni post hoc tests. Correlations were performed using Pearson correlation. ***p < 0.001.

We recently uncovered that disturbed fluid shear stress (FSS) contributes to intima hyperplasia by the induction of endothelial-mesenchymal transition (EndMT)^5^, partially mediated by EZH2^18^. Atheroprotective uniform LSS decreases EZH2 expression at both the gene (2.2-fold, p < 0.001; Fig. 2a) and protein (1.9-fold, p = 0.028; Fig. 2b; Suppl. Fig. 1) level. Uniform laminar shear stress does not change the expression of MAPK7, neither on transcript (Fig. 2c) nor on protein level (Suppl. Fig. 1), however, FSS increases the activity of MAPK7 as indicated by the increased phosphorylation (3.5-fold, p = 0.036, Fig. 2d; Suppl. Fig. 1). Knockdown of EZH2 did not significantly alter MAPK7 transcript expression, whereas MAPK7 activity is increased upon EZH2 reduction (1.9-fold, p = 0.049; Fig. 2d). Moreover, protein expression levels of EZH2 associate with MAPK7 activation (r^2^ = 0.7723, p = 0.021; Fig. 2e) proving evidence of the reciprocity between EZH2 expression levels and MAPK7 activity.

### MAPK7 decreases EZH2 through miRNA-101

**Figure 3:**
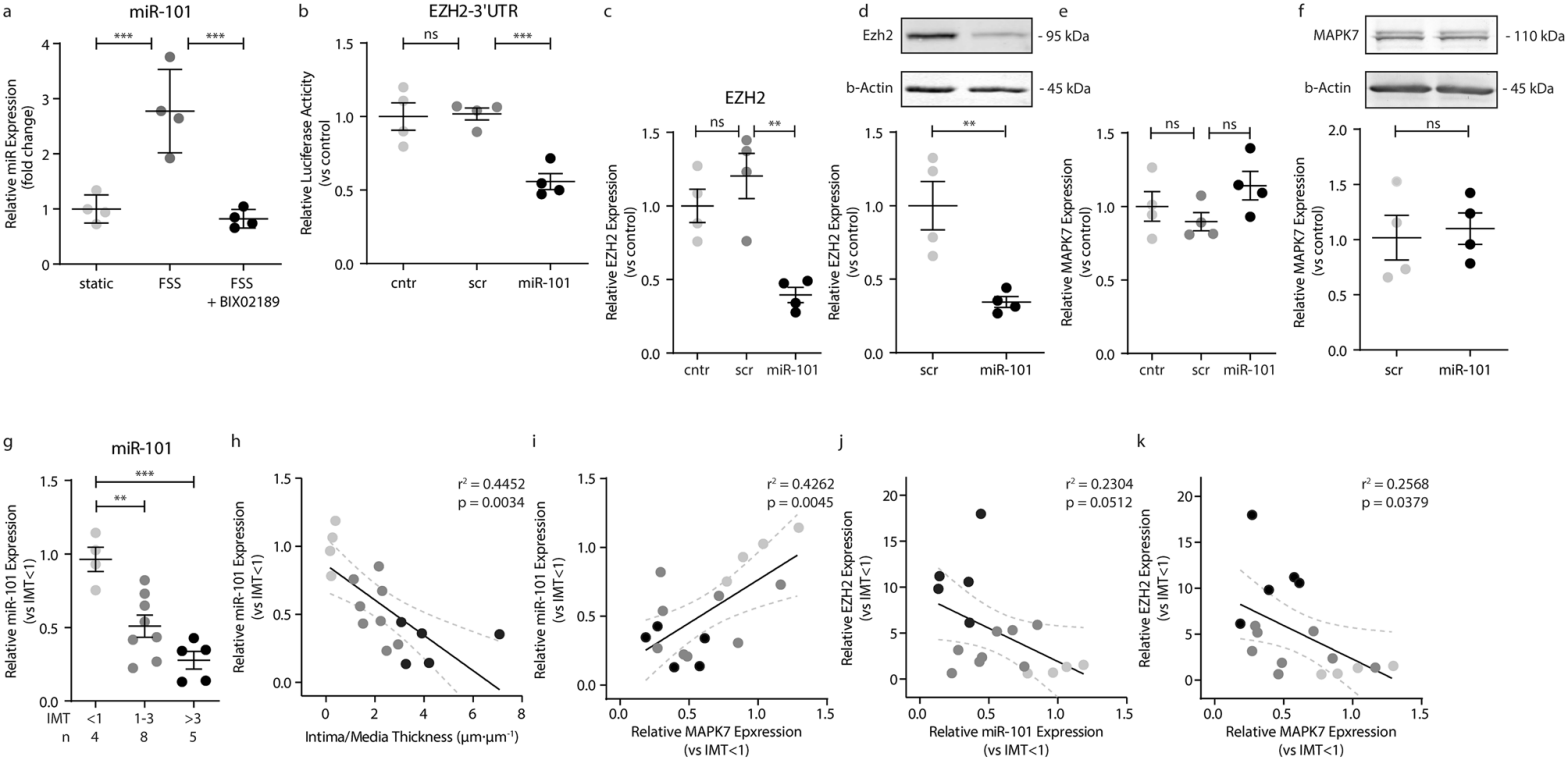
MAPK7 decreases EZH2 through miRNA-101. *MiR-101* expression levels were determined by qPCR in HUVEC exposed to FSS (20 dyne/cm^2^) with or without the MAPK7 inhibitor BIX02189 (10 µM) and normalized to the level of static controls **(a)**. Luciferase reporter binding assays were performed for the 3’UTR of EZH2 in COS7 cells with ectopic expression of miR-101 or scrambled control sequences (scr). Luciferase activity was normalized to non-transfected cells **(b)**. *EZH2* and *MAPK7* expression levels were determined by qPCR in HUVEC with ectopic expression of miR-101 or SCR and normalized to control **(c,e)**. EZH2 and MAPK7 protein levels were determined by western blot in HUVEC with ectopic expression of miR-101 or scrambled control sequences **(d,f)**. *MiR-101* expression levels were determined by qPCR and normalized to IMT < 1 **(g)**. *MiR-101* decreases with increasing IMT **(h)**, and associates with *MAPK7* **(i)**, and tends to associate with *EZH2* **(j) **expression levels. In coronary artery disease, *MAPK7* expression negatively correlates to *EZH2* expression **(k)**. Data is expressed as mean ± S.D. of all individual observations. In vitro experimental data was derived from 4 independent experiments, whereas human in vivo data was derived from n = 4–8 samples per group. Comparisons between 2 groups were performed by Student t-tests and data from multiple groups were analyzed by ANOVA followed by Bonferroni post hoc tests. Correlations were performed using Pearson correlation. *p < 0.05, **p < 0.01, ***p < 0.001.

As MAPK7 decreases EZH2 post-transcriptionally^18^, we investigated whether miRNA-101—a known translational repressor of EZH2 in endothelial cells^22^—is regulated by MAPK7 signaling. FSS increased the expression of miR-101 in a MAPK7-dependent manner (2.8-fold, p < 0.001; Fig. 3a). In luciferase reporter assays, miR-101 binds to the 3’UTR of EZH2, reducing the luminescence signal (1.9-fold, p < 0.001; Fig. 3b). In endothelial cells, ectopic expression of miR-101 decreases EZH2 expression at both the gene (2.6-fold, p = 0.002; Fig. 3c) and protein (2.9-fold, p = 0.008; Fig. 3d; Suppl. Fig. 2) level, whereas miRNA-101 did not significantly alter *MAPK7* gene expression (Fig. 3e) or MAPK7 protein expression level (Fig. 3f; Suppl. Fig. 2). In human coronary artery disease, *miR-101* expression is decreased (p < 0.01, Fig. 3g) and increased disease severity, i.e. increasing IMT, associates with a progressive decrease in *miR-101* (r^2^ = 0.4452, p = 0.003, Fig. 3h). Moreover, the expression level of miR-101 associates with MAPK7 (r^2^ = 0.4262, p = 0.005; Fig. 3i) and tends to associate to EZH2 (r^2^ = 0.2304, p = 0.051; Fig. 3j) in coronary artery disease, where a negative association between MAPK7 and EZH2 expression (r^2^ = 0.2568, p = 0.038; Fig. 3k) is present. Collectively, these data suggest that in coronary artery disease, the reciprocity between MAPK7 activity and EZH2 expression is regulated by miR-101.

### EZH2 regulates DUSP-1 and DUSP-6 expression through miR200a–c

**Figure 4:**
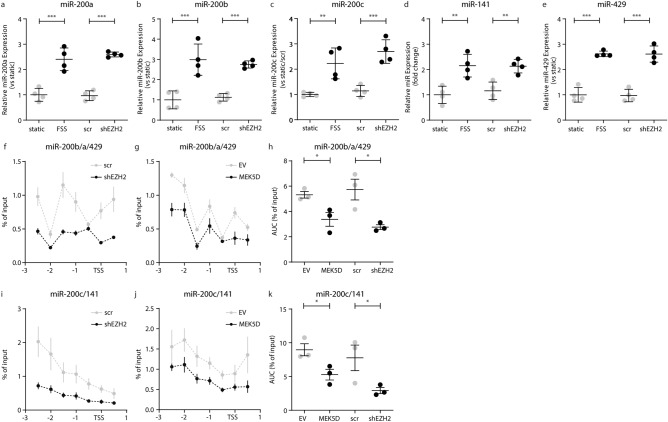
EZH2 regulates DUSP-1 and DUSP-6 expression through miR200a–c. *MiR-200a ***(a)***, miR-200b* **(b)**, *miR-200c* **(c),** *miR-141* **(d)**, and *miR-429* **(e)** expression levels were determined by qPCR in HUVEC exposed to FSS (20 dyne/cm^2^), transduced HUVEC with scr or shEZH2 and normalized to static control cells. H3K27me3 enrichment around the transcription start site (TSS) of the miR-200b/a/429 cluster was determined using ChIP in shEZH2-HUVEC and scr-HUVEC **(f)** and MEK5D-HUVEC and EV-HUVEC **(g)**. H3K27me3 enrichment is shown as area under the curve (AUC) compared to input samples **(h)**. H3K27me3 enrichment at the around the TSS of miR-200c/141 cluster was determined using ChIP in scr-HUVEC and shEZH2 **(i)** and in MEK5D-HUVEC and EV-HUVEC **(j)**. H3K27me3 enrichment is shown as AUC compared to input samples **(k)**. Data is expressed as mean ± S.D. of all individual observations. Gene expression data were obtained from 4 independent experiments. Data from ChIP experiments were obtained from three independent experiments. All data was analyzed by ANOVA followed by Bonferroni post hoc tests. *P < 0.05, **P < 0.01, ***P < 0.001.

EZH2 expression determines the level of MAPK7 activity. However, EZH2 is a transcriptional repressor that cannot directly regulate the activity of a kinase. MAPK7 activity is regulated by the Dual Specificity Phosphatases (DUSP)-1 and DUSP-6^21^, yet a reduction in EZH2 expression is associated with a decreased expression of DUSP-1 and DUSP-6^23,24^. Therefore, we investigated alternative mechanisms that might decrease DUSP expression upon the reduction of EZH2. In silico analysis, using Targetscan.org^25^ to identify microRNAs that putatively target DUSP-1 and DUSP-6 was followed by cross-referencing for EZH2 or H3K27Me3 interactions in endothelial cells and mesenchymal cells using the genome browser (ENCODE Histone Modifications track set, genomebrowser.org for HUVEC, human microvascular endothelial cells (HMEC), normal human dermal fibroblasts (NHDF) and normal human lung fibroblasts (NHLF)) and putatively identifies the microRNA-200 family (miR-200a, miR-200b, miR-200c, miR-141 and miR-429) as regulators of DUSP-1 and DUSP-6 with different ChIP signal between endothelial cells (low signal) and mesenchymal cells (high signal). Therefore, we investigated if the expression of the miR-200b/a/429 cluster on chromosome 1 and the miR-200c/141 cluster on chromosome 12 are under control of EZH2. Uniform LSS increased the expression of all microRNAs of the miR-200b/a/429 and miR-200c/141 clusters (Fig. 4a–e). Moreover, knockdown of EZH2 similarly increased the expression of microRNAs in these clusters (Fig. 4a–e).

The EZH2-induced trimethylation of lysine 27 on histone 3 (H3K27Me3) is present in the promoter regions of the miR-200b/a/429 and miR-200c/141 clusters (Fig. 4f,i). Knockdown of EZH2 in endothelial cells reduced the level of H3K27Me3 at these gene regions (Fig. 4f,i), and the loss of this repressive histone mark coincided with increased expression of miR-200a–c, miR-141 and miR-429. In endothelial cells with constitutively active MAPK7 signaling (MEK5D), the abundance of H3K27Me3 is decreased at the promoter regions of miR-200b/a/429 (1.6-fold, p = 0.034; Fig. 4g,h) and miR-200c/141 (1.9-fold, p = 0.035; Fig. 4j,k), implying that MAPK7 activity increases the expression of miR-200 family members through a decrease in EZH2-mediated gene silencing.

**Figure 5:**
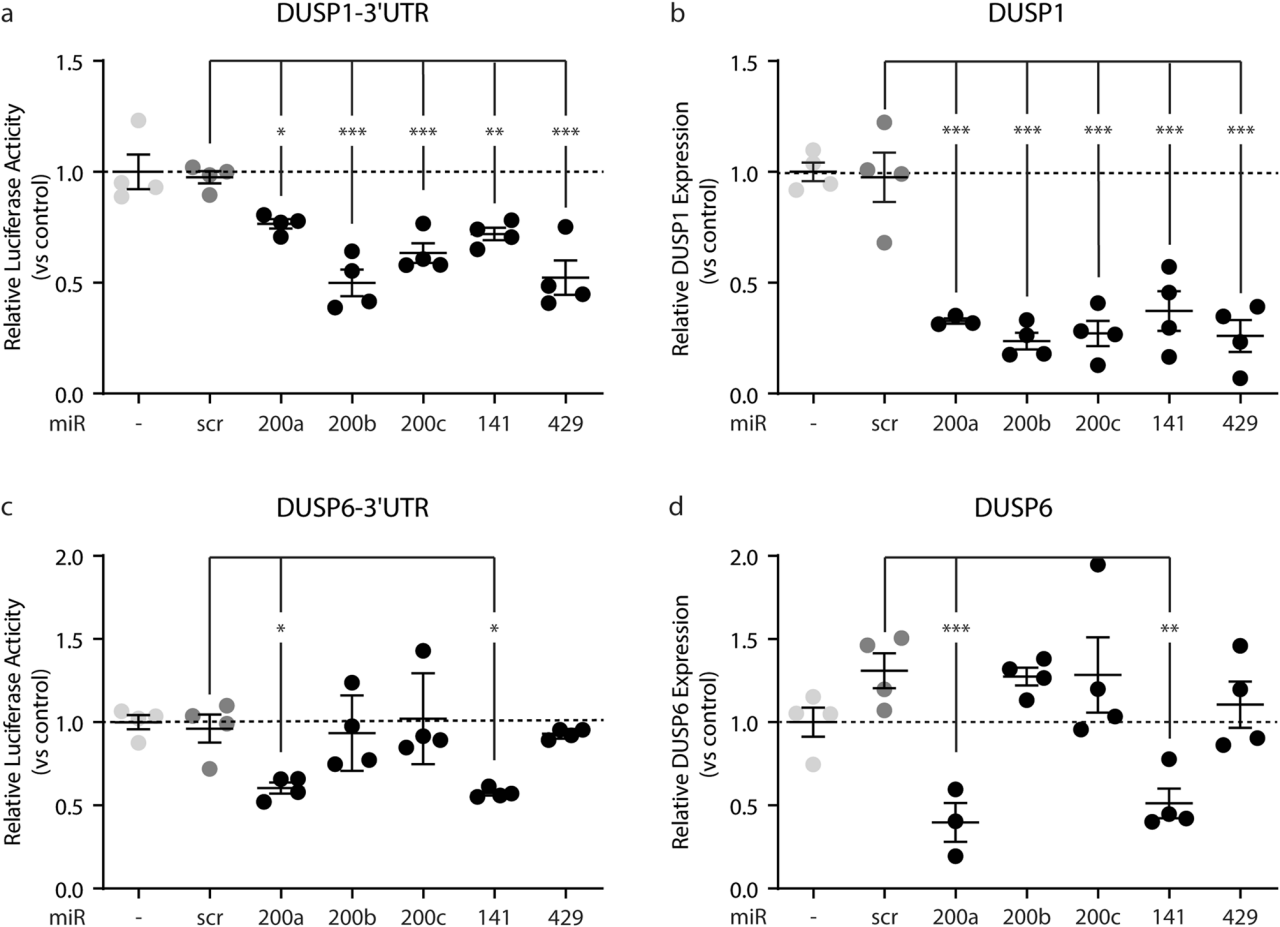
miRNA-200 family members regulated DUSP-1 and DUSP-6 expression. Luciferase reporter assays for microRNA binding were performed for the 3’UTR of DUSP-1 in COS7 cells with ectopic expression of miR-200a, -200b, -200c, -141, -429 or SCR **(a)**. *DUSP-1* expression levels were determined by qPCR in HUVEC with ectopic expression of miR-200b, miR-200a, miR-429, miR-200c, miR-141 or scr, normalized to control. All miRNA-200 family members decrease *DUSP-1* expression in HUVEC **(b)**. Luciferase reporter assay identified miR-200a and miR-141 to target DUSP-6 **(c)** and ectopic expression of miR-200a or miR-141 decreases *DUSP-6* expression in HUVEC **(d)**. Data is expressed as mean ± S.D. of all individual observations. Gene expression data and Luciferase activity measurements were obtained from 4 independent experiments. All data was analyzed by ANOVA followed by Bonferroni post hoc tests. *P < 0.05, **P < 0.01, ***P < 0.001.

In luciferase reporter assays, all miR-200 family members were able to bind to the 3’UTR of DUSP-1 (Fig. 5a), but only miR-200a and miR-141 were able to bind the 3’UTR of DUSP-6 (Fig. 5c). Corroboratively, exogenous expression of all miR-200 family members in endothelial cells decreased DUSP-1 expression (Fig. 5b), whereas only miR-200a and miR-141 decreased the expression of DUSP-6 (Fig. 5d). Collectively, these data imply that the activation of MAPK7 by uniform LSS decreases the expression of DUSP-1 and DUSP-6 expression via the EZH2-dependent regulation of miR-200b/a/429 and miR-200c/141 expression.

### Pharmacological inhibition of DUSP activity does not alter MAPK7 activity and EZH2 expression

**Figure 6:**
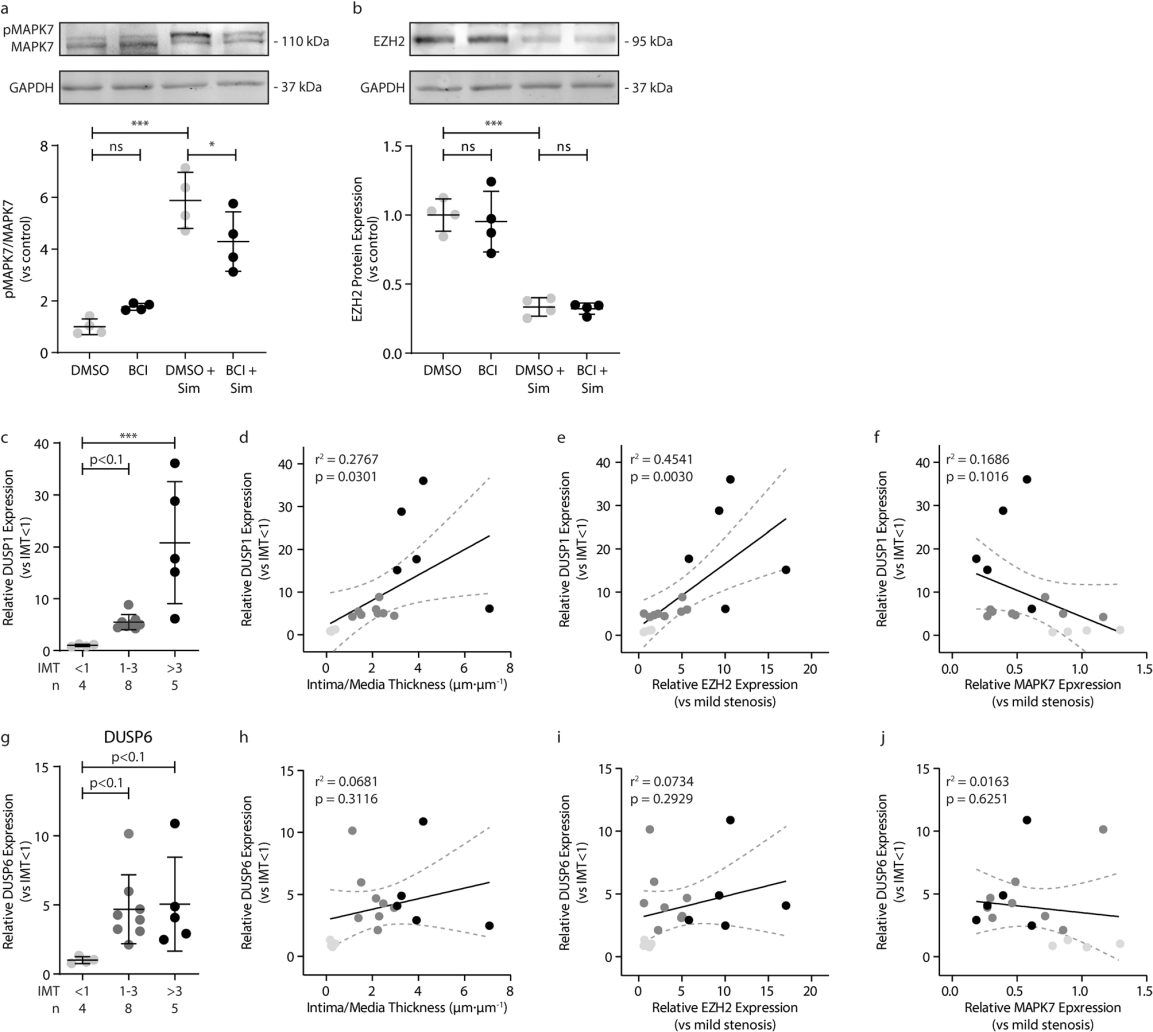
Inhibition of DUSP activity by BCI does not affect MAPK7 activity or EZH2 expression. MAPK7 **(a)** and EZH2 **(b)** protein expressions were determined using western blotting, in HUVEC treated with 5 µM of the DUSP-1/6 small molecule inhibitor BCI or co-treated with BCI and 10 µM Simvastatin for 24 h. Data was normalized to vehicle control cells. *DUSP-1* expression levels were determined in human coronary artery disease samples by qPCR and normalized to IMT < 1 **(c)**. *DUSP-1* expression increases with increasing IMT **(d)** and is associated to EZH2 expression levels **(e)**. *DUSP-1* levels tend to show a negative correlation with *MAPK7* levels in coronary artery disease **(f)**. *DUSP-6* expression levels were determined by qPCR and normalized to IMT < 1 **(g)**. *DUSP-6* expression is elevated in coronary artery disease but does not associate to the severity of disease **(h)**, the level of *EZH2* expression **(i)**, nor the level of *MAPK7* expression **(j)**. Sim = Simvastatin (10 µM). Data is expressed as mean ± S.D. of all individual observations. Cell culture data was obtained from 4 independent experiments, whereas human in vivo data was derived from n = 4–8 samples per group. Data from multiple groups were analyzed by ANOVA followed by Bonferroni post hoc tests. Correlations were performed using Pearson correlation. **p < 0.01, ***p < 0.001.

We investigated if the pharmacological inhibition of DUSP-1 and DUSP-6 activity in endothelial cells would activate MAPK7 signaling and decrease the expression of EZH2. BCI-treated endothelial cells modestly increased MAPK7 phosphorylation (1.7-fold; Fig. 6a; Suppl. Fig. 3), albeit not statistically significant (p = 0.202), and did not affect EZH2 expression (Fig. 6b; Suppl. Fig. 3). The addition of Simvastatin – a known activator of MAPK7 signaling^15^ – did increase increased MAPK7 activation (5.8-fold, p < 0.001; Fig. 6a; Suppl. Fig. 3) and decreased EZH2 protein expression (3.0-fold, p < 0.001; Fig. 6b; Suppl. Fig. 3). The addition of BCI to simvastatin-treated endothelial cells did not increase the levels of MAPK7 activation nor decrease the protein expression of EZH2 further. Rather, the addition of BCI reduced the simvastatin-induced activity of MAPK by ~ 27% (p = 0.033; Fig. 6a; Suppl. Fig. 3).

In human coronary artery disease, *DUSP-1* expression is increased in advanced lesions (IMT > 3, p < 0.001, Fig. 6c) and increasing IMT associates with increased *DUSP-1* expression (r^2^ = 0.2767, p = 0.0301; Fig. 6d). Moreover, the increase in *DUSP-1* expression associates with increased *EZH2* expression in coronary artery disease (r^2^ = 0.4541, p = 0.0030; Fig. 6e) and the increase in *DUSP-1* expression tends to associate with decreased *MAPK7* expression (r^2^ = 0.1686, p = 0.1016; Fig. 6f), although not significantly. Also, *DUSP-6* seems to be increased in coronary artery disease (p < 0.1, Fig. 6g), albeit not significantly. The apparent increase in *DUSP-6* expression does not significantly associate with increasing IMT (r^2^ = 0.0681, p = 0.3116; Fig. 6h), nor do the expression levels of DUSP-6 significantly associate with *EZH2* (r^2^ = 0.0734, p = 0.2929; Fig. 6i) and *MAPK7* expression (r^2^ = 0.0163, p = 0.6251; Fig. 6j) in the coronary artery tissue.

### Ectopic expression of miRNA-101, miRNA-141 and miRNA-200a inhibits endothelial dysfunction and EndMT

**Figure 7:**
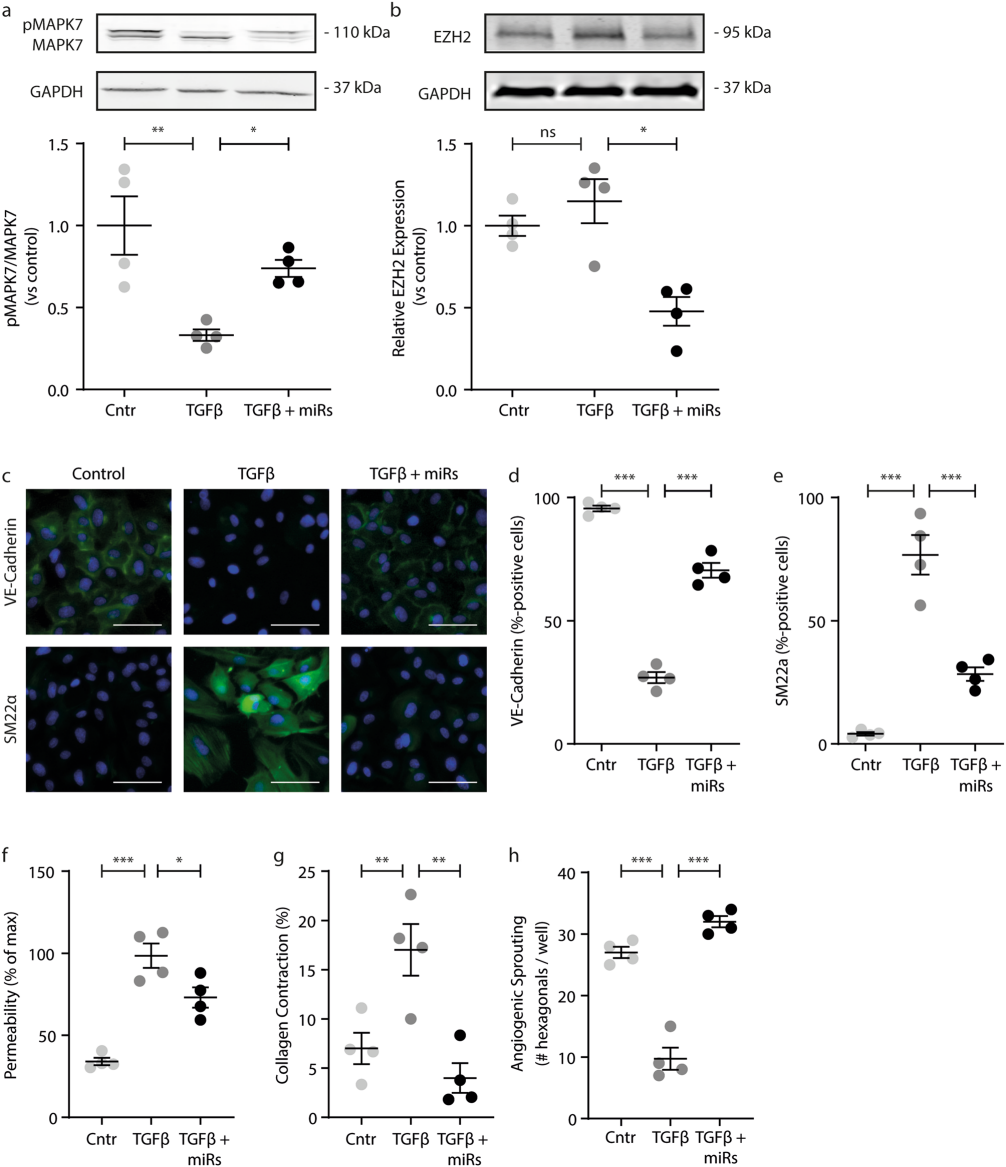
Ectopic expression of miRNA-101, miRNA-141 and miRNA-200a inhibits endothelial dysfunction and EndMT. MAPK7 **(a)** and EZH2 **(b)** protein expressions were determined using western blotting, in HUVEC treated with 10 ng/ml TGFb1 with ectopic expression of miRs-101, -200a and -141 and normalized to untreated control cells. The expression of VE-Cadherin **(c,d)** and SM22a **(c,e)** were assessed by immunofluorescence and quantified using TissueFaxs analyses. Endothelial cell permeability was assessed using transwell FITC-dextran leakage **(f)** and collagen gel contraction **(g)** was assessed as a mesenchymal cell function. The angiogenic sprouting behavior of endothelial cells was assessed using the Matrigel assay **(h)**. Data is expressed as mean ± S.D. of all individual observations. Cell culture data was obtained from 4 independent experiments. Data from multiple groups were analyzed by ANOVA followed by Bonferroni post hoc tests. *p < 0.05, **p < 0.01, ***p < 0.001. Scale bar = 20 µm.

As coronary artery disease is associated with EndMT^1,26^, we investigated if the ectopic expression of miR-101 or miR-200 family members could preclude EndMT. Endothelial cells transfected with only a single microRNA were susceptible to TGFβ1-induced EndMT (data not shown), however, when miR-101, miR-200a and miR-141 were transfected in combination, endothelial cells increased their MAPK7 activity (Fig. 7a; Suppl. Fig. 4) and showed reduced expression levels of EZH2 (Fig. 7b; Suppl. Fig. 4). Corroborating the protective effects of MAPK7 signaling in the preclusion of EndMT^5^, TGFβ1 stimulation did not decrease VE-Cadherin expression (Fig. 7c,d) nor induce the expression of the mesenchymal marker protein SM22α (Fig. 7c,e) in endothelial cells transfected with miRs-101/200a and -141. Moreover, the ectopic microRNA expression reduced the TGFβ1-induced increase in endothelial permeability by ~ 40% (Fig. 7f) and precluded the TGFβ1-induced collagen contraction (Fig. 7g) – two functional adaptations associated with EndMT – and maintained the endothelial angiogenic sprouting capacity (Fig. 7h).

## Discussion

**Figure 8:**
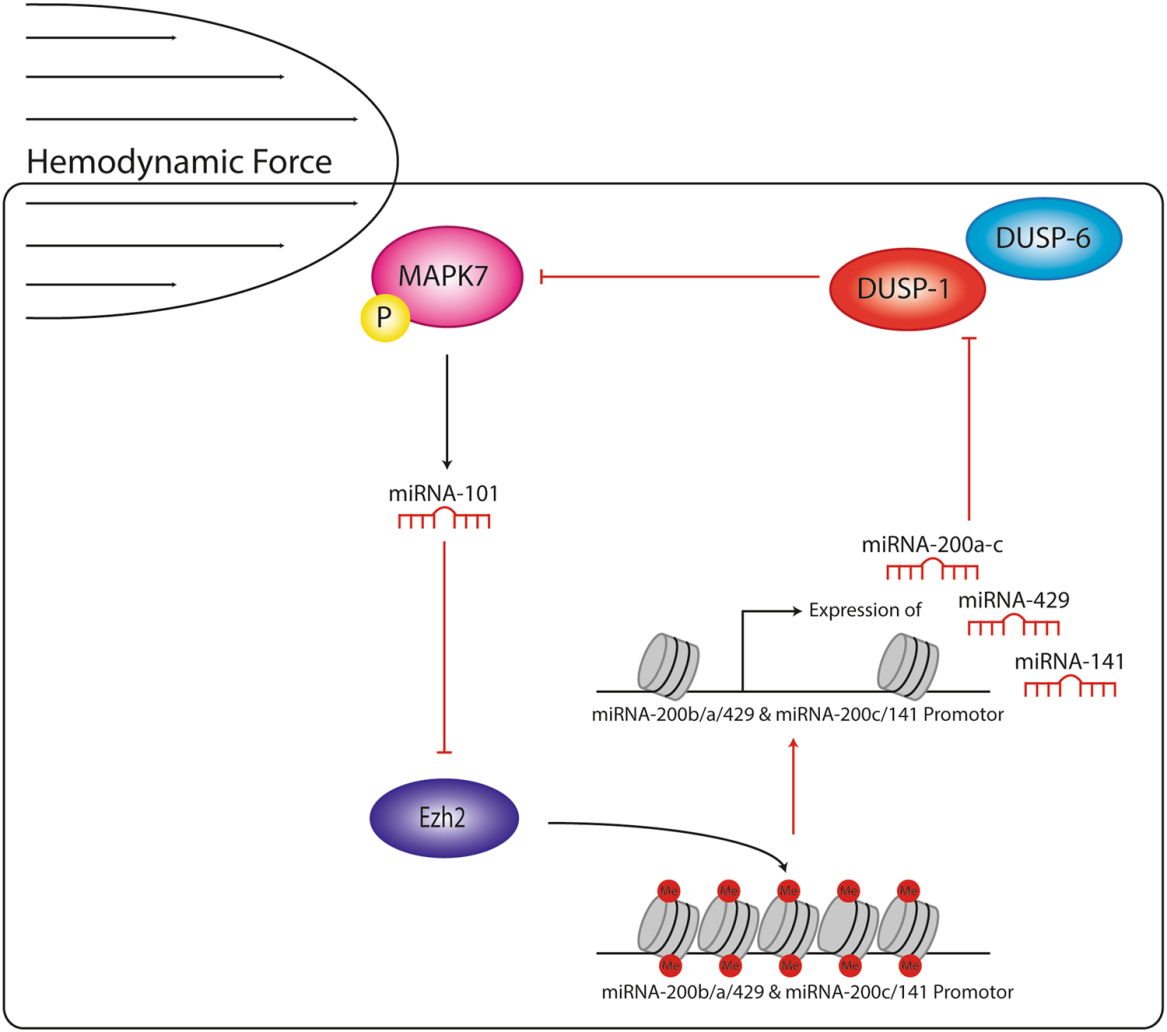
Reciprocal signaling between MAPK7 activity and EZH2 expression in endothelial cells. This reciprocity is regulated by the MAPK7-induced silencing of EZH2 expression by miR-101 and the EZH2-mediated silencing of the miR-200 family, which increases DUSP-1 and DUSP-6 expression and inhibits MAPK7 activation. The reciprocity between MAPK7-EZH2 might reflect an autoregulatory feedback loop in endothelial cells that ensures endothelial homeostasis.

In this study, we show that reciprocity exists between the atheroprotective MAPK7 activation and the expression of histone methyltransferase EZH2 in endothelial cells. The reciprocity is regulated by the MAPK7-induced silencing of EZH2 expression by miR-101 and the EZH2-mediated silencing of the miR-200 family, which increases DUSP-1 and DUSP-6 expression and inhibits MAPK7 activation (Fig. 8). The reciprocity between MAPK7-EZH2 might reflect an autoregulatory feedback loop in endothelial cells that ensures endothelial homeostasis. As such, disturbances in this reciprocity leading to increased EZH2 expression can induce endothelial dysfunction and EndMT. In contrary artery disease—a condition associated with EndMT^1,26^—the reciprocity between MAPK7 and EZH2 is disturbed, resulting in elevated expression of DUSP-1 and EZH2 and the decreased expression of MAPK7. Restoring the reciprocity by ectopic expression of miR-101/200a/429 precludes EndMT and might offer therapeutic benefit in coronary artery disease.

EndMT contributes to intimal hyperplasia during coronary artery disease^1–5^, wherein MAPK7 signaling plays a protective role^5,16^. EndMT can be induced by hypoxia, inflammatory and fibrogenic signaling ^6,27^. Transforming growth factor beta (TGFβ) induces EndMT canonically through the activation of downstream mediators Smad2/3, which culminates in the activation of the EndMT transcription factors Snail, Slug and Twist1 ^6^. MAPK7 inhibits EndMT^5,28^, potentially via the increased expression of inhibitory Smad7^29,30^ and ID proteins^31^ or the repression of TGF control elements in the promoter region of mesenchymal genes^32^. Yet, during intimal hyperplasia the signaling activity of MAPK7 is rapidly lost^28^.

DUSP-1 and DUSP-6 expression levels are elevated in a number of cardiovascular diseases and DUSP-1 deficient mice are protected from atherosclerosis development^33,34^. The elevated expression of DUSPs might explain the loss in protective MAPK7 signaling activity during coronary artery disease. Yet we could not substantiate this hypothesis as pharmacological inhibition of DUSP-1/6 in our experiments did not activate MAPK7 signaling, which may also depend on the availability of ATP and scaffolding proteins, and the activity of upstream MAPK kinases^35^. Besides, the regulation of MAPK7 activity by DUSPs is more complex than direct inactivation. Indeed, DUSP activity is greatly affected by posttranslational modifications^36–40^. For instance, DUSP activity is enhanced by its acetylation^37,38^ or phosphorylation^36^, whereas its oxidation reduces its activity^39,40^. In our experiments, we did not investigate the posttranslational modifications of DUSP1, and in the absence of a response to the small molecule BCI, it is tempting to speculate that the prerequisites for DUSP1 activity were not met in our cell culture model. Moreover, the family of DUSPs contains over twenty members with overlapping substrate specificity^41^, that are not all inhibited by the addition of BCI^42^. It is therefore conceivable that alternative DUSPs maintain the inhibition of MAPK7 activity in the presence of BCI as a compensatory mechanism. Nonetheless, the expression of DUSP-1 is associated with an increasing IMT, and a decreased expression of MAPK7 in coronary artery disease.

The expression of DUSP-1 and -6 is associated with high expression of EZH2 in various oncology’s^23,24^, albeit by a currently unknown mechanism. We found that EZH2 silences the expression of the microRNA-200 family, which posttranscriptionally regulate the expression of DUSP-1 and DUSP-6. The loss of EZH2 expression by fluid shear stress therefore might increase the expression of miR-200 family members and decrease the expression of the DUSPs culminating in atheroprotective MAPK7 activation. Interestingly, the endothelial cell-specific overexpression of miR-200b precludes EndMT and alleviates diabetic cardiomyopathy in mice^43^. In coronary artery disease, EZH2 expression levels are elevated and high EZH2 expression is associated with endothelial dysfunction^18,19^. In combination, our current data might explain these observations and unifies them into a single mechanism, linking endothelial mechanotransduction to the epigenetic regulation of MAPK7 activity, potentially through DUSP-1 and DUSP-6. This double negative feedback loop might resemble a sensitive autoregulatory mechanism that ensures endothelial homeostasis, which when disturbed culminates in EndMT and possibly coronary artery disease.

It should be noted here, that the downstream effects of EZH2 and MAPK7 on atherogenesis may be much broader than the reciprocal regulation of their expression and activation. Indeed, in earlier work we used RNA sequencing and found that high EZH2 expression coincides with endothelial cell proliferation and RNAi-mediated silencing of EZH2 results in endothelial quiescence^18^. Using a similar approach, others reported that high EHZ2 associates with a reduced angiogenic potential and inflammatory activation of endothelial cells^19^. Similarly, the endothelial-specific genetic deletion of MAPK7 aggravates atherogenesis by the induction of endothelial cell inflammatory activation and loss of atheroprotective NO synthesis^16^ and the induction of EndMT^5^. Thus, in future perspective it would be of high interest to validate and extend our current findings in atherosclerosis-prone mouse models (e.g. APOE-deficient or LDLR-deficient mice on a cholesterol-rich diet) wherein MAPK7 and EZH2 expression can be perturbed specifically in the endothelium. Such studies would benefit from the use of transcriptomic, proteomic or “multi-omics” approaches to unravel the multitude of targets and the principle downstream pathways EZH2 and MAPK7 regulate in the atherosclerotic endothelium.

### Study limitations

We acknowledge that our study is not without limitations. First, we only included unique 17 coronary artery samples from 10 subjects, which may be a limited number if confounders as hypertension, diabetes or smoking status are to be analyzed. In our study, we chose to stratify these coronary artery samples based on IMT (as a surrogate marker of disease severity) to investigate if the expression of MAPK and EZH2 are “disease-state” specific, and thereby ignored interindividual variation, which may be caused by common risk factors^44,45^ or genetic susceptibility^46,47^. In our study, mean age, hypertension, diabetes and smoking status did not differ between stratified groups and no subject was overrepresented in any group. Using this approach, we show that there is a disbalance in MAPK7 activity and EZH2 expression in coronary artery disease and that this disbalance is perturbed with increasing IMT. In future perspective, it would be highly interesting to investigate how common risk factors affect the disbalance between MAPK7 activity and EZH2 expression in larger cohort studies. In addition, and since polymorphisms in MAPK7^48–50^ and EZH2^51–54^ are identified, it would be highly interesting to investigate whether these polymorphisms associate to a higher cardiovascular risk.

Second, transcriptional expression was performed on of whole artery sections, meaning that the observed differences in MAPK7 and EZH2 expression are not necessarily derived from endothelial cells, but may reflect a significant change in other cell numbers, e.g. inflammatory and smooth muscle cells. Indeed, the cellular composition of a coronary artery lesion is dynamic and may change with disease progression^55,56^. Although MAPK7 expression appears endothelial cell-restricted in porcine and mouse arteries^28^, EZH2 is expressed by a plethora of cells. In vivo validation of our current findings by localization techniques such as double immunofluorescence would have to be performed in order to show endothelial specificity of the reported results. Moreover, the endothelial cell-specific deletion of MAPK7 and EZH2 in atherosclerosis-prone mice would further emphasize their relevance to atherogenesis in future perspective.

Third, for the mechanistic experiments detailed here, we used HUVEC rather than primary human coronary artery endothelial cells (HCAEC), and their distinct origin might interfere on our observations. Although the endothelium harbors a larger heterogeneity in vivo between endothelial cells from distinct vascular beds^57,58^, cultured endothelial cells rapidly lose the expression of vascular bed-specific markers^57–60^. HUVEC maintain expression of generalized endothelial function in cell culture, including shear stress responsiveness^5,61^. Hence, HUVEC are commonly used for mechanistic studies of endothelial cell behavior^62^. Moreover, HUVEC and HCAEC show similar in vitro responses to LSS stimulation^61^.

## Conclusion

To summarize, in endothelial cells there is reciprocity between MAPK7 activity and the expression of EZH2. This reciprocity is regulated in part by a complex mechanism involving microRNAs and the regulation of phosphatase activity (Fig. 8). Dysregulation in the reciprocity between MAPK7 activation and EZH2 expression is associated to the induction of EndMT and the severity of coronary artery disease. These insights contribute to a better understanding of the molecular and epigenetic mechanisms that underlie endothelial homeostasis, the induction of EndMT during coronary artery disease and might represent a new target for therapy.

## Materials and methods

### Human coronary artery samples

Seventeen unique human coronary artery samples were obtained from autopsy specimens from 10 patients (age 59.1 ± 2.6 years, range 39–69) that died from an acute coronary episode at the Heart Institute (InCor), Sao Paulo, Brazil. Hypertension was present in 9 subjects, and diabetes in 6. Five individuals were active smokers. Coronary artery samples were stratified based on their respective intima-media thickness (IMT) prior to further analyses. Stratified sample groups never contained more than one sample per patient. Next-of-kin gave informed consent and the investigation was performed according to institutional guidelines and approved by the Institutional CAPPesq Ethics Committee (InCor, Sao Paulo #SDC 3723/11/141 and #CAPPesq 482/11) and the Declaration of Helsinki^28^. All experimental protocols were approved by the Institutional CAPPesq Ethics Committee (InCor, Sao Paulo, Brazil). During necropsy each dissected coronary artery was fixed in neutral-buffered formalin with constant perfusion at a quasi-normal perfusion pressure before paraffin embedding.

### Determination of intima-media thickness

Four micron-thick sections were prepared from human coronary artery samples and deparaffinized using Xylol and rehydrated using a series of EtOH solutions of decreasing concentration. Samples were stained in Verhoeff’s solution (92 mM hematoxylin, 137 mM FeCl_3_, 27 mM KI, 4 mM I_2_ in 55% EtOH) at room temperature for 1 h. Samples were differentiated in FeCl_3_ (123 mM in dH_2_O) for 1 min and treated with Sodium Thiosulphate (316 mM in dH_2_O) at room temperature for 1 min. Samples were dehydrated using increasing concentrations of EtOH and cleared in 100% xylene. Samples were mounted in Permount resinous mounting medium. The intimal thickness was determined as the distance between the inner elastic lamina and the lumen, and the medial thickness was determined by measuring the distance between the inner elastic lamina and the outer elastic lamina at ten sites within one sample. Intimal/Medial thickness was calculated by dividing the average intimal thickness by the average medial thickness^28^.

### Endothelial cell culture and uniform laminar shear stress experiments

Human umbilical vein endothelial cells (HUVEC, Lonza #C2519) were cultured in endothelial cell culture medium (ECM) as described previously^5,63^. EndMT was induced by the addition of 10 ng/ml TGFβ1 to the culture medium as described before^5,64^. For shear stress experiments, HUVEC (60 000 cells/cm^2^) were seeded on 0.1% gelatin-coated µ-Slides I 0.4 Luer (Ibidi GmbH, Martinsried, Germany) and allowed to adhere under standard culture conditions overnight. Slides with a confluent endothelial cell monolayer were exposed to uniform laminar shear stress (20 dyne/cm^2^) for 24 h. Where indicated, 10 µM of the small molecule inhibitor of MAPK7 (BIX02189, SelleckChem, Munich, Germany)^14^, 5 µM of the small molecule inhibitor of DUSP-1/6 (BCI, Axon Medchem, Groningen, The Netherlands) or 10 µM simvastatin (SelleckChem, Munich, Germany)^65^ was applied.

### Viral transduction of endothelial cells

pLKO.1-shEZH2 and pLKO.1-SCR were kindly provided by Prof.dr. J.J. Schuringa (dept. Hematology, UMCG). HEK293 cells were co-transfected with pLKO.1-shEZH2 or pLKO.1-SCR, pVSVG (envelope plasmid) and pCMV-R8.91 (gag-pol 2nd generation packaging plasmid) using Endofectin-Lenti (Gene Copoeia, Rockville, MD, USA). At 48- and 72-h post-transfection, viral supernatants were collected.

A retroviral construct encoding the constitutively active rat MEK5-α1 (pBabePuro-MEK5D) and empty vector controls were kindly provided by Prof.dr. M. Schmidt (Dept. Dermatology, University Würzburg, Germany). Retroviral transduction of HUVEC was performed as detailed before^66^. In brief, virus-producing Phoenix cells were cultured until 70% confluency, after which basal medium was replaced by ECM after which viral supernatants were collected twice at 24 h intervals.

Viral supernatants were supplemented with polybrene (6 µg/ml; Sigma, St.Louis, MO) and applied to 30% confluent HUVEC for two consecutive rounds of 24 h exposure. Transduced HUVECs were passaged twice, and transduced cells were selected by puromycin (4 µg/ml; Invitrogen, Carlsbad, CA, USA).

### MicroRNA transfections in endothelial cells

HUVEC or COS7 cells were seeded in antibiotic free medium at a density of 20,000/cm^2^. Cells were transfected with 50 pmol of microRNA mimics (miR-101 (#PM11414), miR-200a (#PM10991), miR-200b (#PM10492), miR-200c (#PM11714), miR-141 (#PM10860), miR-429 (#PM10221) or scrambled control (#AM17110, all Ambion/Life Technologies, Carlsbad, CA) using the siRNA reagent system (Santa Cruz, #sc-45064, Santa Cruz, CA) according to manufacturer’s instructions.

### Immunofluorescence

Immunofluorescence analysis was performed for the endothelial cell marker VE-Cadherin (R&D #9381, Minneapolis, MN) and the mesenchymal cell marker SM22α (Abcam #14106, Cambridge, UK) as previously described^64^.

### Immunoblotting

Cells were harvested in RIPA buffer (Thermo Fisher Scientific, Waltham, MA) supplemented with 1% v/v protease inhibitor cocktail (Sigma Aldrich, St Louis, MO) and 1% v/v phosphatase inhibitor cocktail (Sigma Aldrich, St Louis, MO). Samples were sonicated and protein concentration was determined with a DC protein assay (Bio-Rad, Hercules, CA). Equal amounts of protein were separated by electrophoresis on 10% polyacrylamide gels after which proteins were blotted onto nitrocellulose membranes using the semi dry Transblot Turbo system (Bio-Rad, Hercules, CA). Membranes were trimmed to conserve antibodies and blocking reagent, and were blocked with Odyssey Blocking buffer (Li-COR Biosciences, Lincoln, NE) at RT for 1 h, and incubated with antibodies against β-actin (1:2000, Cell Signaling, Danvers, MA, USA), EZH2 (1:1000, Cell Signaling, Danvers, MA, USA), MAPK7 (1:1000, Merck Millipore, Billerica, MA, USA), MKP-1(DUSP-1, 1:1000, Abcam #195261) or MKP-3 (DUSP-6, 1:500, Santa Cruz, #sc377070) at 4 C overnight. Membranes were washed in TBS Tween (0.1%) and developed using IrDye-conjugated antibodies to rabbit IgG (1:10,000, #926-68021), mouse IgG (1:10,000, # 926-32210, both Li-COR Biosciences) or AP-conjugated antibodies to rabbit IgG (1:2000, #7054S, Cell Signaling) at RT for 1 h. Protein detection was done using the Odyssey Infrared Imaging System (Li-COR Biosciences). The development of AP-conjugated antibodies, membranes were incubated with AP-detection buffer (100 nM NaCl, 100 mM Tris, 50 mM MgCl_2_, pH 9.5) supplemented with nitro-blue tetrazolium chloride (NBT) (330 µg/mL) and 5-bromo-4-chloro-3'-indolyphosphate p-toluidine salt (BCIP) (165 µg/mL). Densitometry analysis was performed using Totallab 120 (Nonlinear Dynamics, Newcastle upon Tyne, England). Unprocessed images of individual immunoblots used for quantification are available in the online data supplement.

### RNA isolation and transcript analysis

Sections of whole arterial thickness were deparaffinated using xylol and rehydrated prior to homogenization in TRIzol (Invitrogen Corp, CA, USA). Cell cultures were lysed directly in TRIzol. RNA was isolated using the TRIzol reagent according to the manufacturer's protocol. RNA concentration and purity were assessed using UV spectrometry (Nanodrop 1000, Thermo Scientific MA, USA) and RNA integrity validated on 1% agarose gels. For gene expression analysis, cDNA synthesis was performed using RevertAid™ First Strand cDNA Synthesis Kit (Thermo Scientific, MA, USA), according to the manufacturer's protocol. For microRNA transcript analysis, 10 ng of total RNA was reversely transcribed using the ABI Taqman microRNA reverse transcription kit (#4366597, ThermoFisher Scientific) according to manufactures instructions using 1.0 µM microRNA-specific stemloop primers (Table 1). For all transcript analyses, the cDNA was amplified on a VIIA7 thermal cycling system (Applied Biosystems, Carlsbad, CA) in a reaction containing 0.6 µM primers (Table 2) using SYBR Green chemistry (Bio-Rad, VA, USA). Cycle threshold (C_T_) values for individual reactions were determined and normalized against GAPDH/ACTB (gene transcript analysis) or RNU6 (microRNA transcript analysis). All cDNA samples were amplified in triplicate. Relative expression was calculated using the ΔC_T_ method. Data are presented as fold change compared with control.

**Table 1:** Primer sequences for microRNA expression analysis.

Gene	Sequence
*miR-101*	Stem loop: GTCGTATCCAGTGCAGGGTCCGAGGTATTCGCACTGGATACGACTTCAGTTA Sense: TGCGGTACAGTACTGTGAT
*miR-141*	Stem loop: GTCGTATCCAGTGCAGGGTCCGAGGTATTCGCACTGGATACGACCCATCTTTAC Sense: TGCGGTAACACTGTCTG
*miR-200a*	Stem loop: GTCGTATCCAGTGCAGGGTCCGAGGTATTCGCACTGGATACGACACATCGTT Sense*:* TGCGGTAACACTGTCTGGT
*miR-200b*	Stem loop: GTCGTATCCAGTGCAGGGTCCGAGGTATTCGCACTGGATACGACTCATCATTAC Sense: TGCGGTAATACTGCCTG
*miR-200c*	Stem loop: GTCGTATCCAGTGCAGGGTCCGAGGTATTCGCACTGGATACGACCCAAACACTG Sense: TGCGGCGTCTTACCCAG
*miR-429*	Stem loop: GTCGTATCCAGTGCAGGGTCCGAGGTATTCGCACTGGATACGACACGGTTTTAC Sense: TGCGGTAATACTGTCTG
*U6*	Stem loop: GTCGTATCCAGTGCAGGGTCCGAGGTATTCGCACTGGATACGACAAAAATATGG Sense: TGCGGCTGCGCAAGGATGA
*U24*	Stem loop: GTCGTATCCAGTGCAGGGTCCGAGGTATTCGCACTGGATACGACTGCATCAGCG Sense: TGCGGTGCAGATGATGTAA
	Antisense: GTGCAGGGTCCGAGGT

**Table 2:** Primer sequences for gene expression analysis.

Gene	Sequence
*DUSP-1*	Sense: TGGGTACATCAAGTCCATCTGA Antisense: GCAAAAAGAAACCGGATCAC
*DUSP-6*	Sense: GACGCTCGCTGTTTGTATCC Antisense: GACTCAGCCTCGCACACC
*EZH2*	Sense: GCGAAGGATACAGCCTGTGCACA Antisense: AATCCAAGTCACTGGTCACCGAAC
*GAPDH*	Sense: AGCCACATCGCTCAGACAC Antisense: GCCCAATACGACCAAATCC
*MAPK7*	Sense: CCTGATGTCAACCTTGTGACC Antisense: CCTTTGGTGTGCCTGAGAAC

### 3’UTR binding assays

3’UTR fragments were isolated, purified and cloned in the psiCKECK-2 reporter vector as described previously^64^. Specific primers for the *EZH2*-3’UTR (sense 5’-CATCTGCTACCTCCTCCCCC-3’, antisense 5’-GACAAGTTCAAGTATTCTTT-3’), *DUSP-1*-3’UTR (sense 5’-AAGGCCACGGGAGGTGAGGC-3’, antisense 5’-CAATAGAAATGCCATAATTT-3’), and *DUSP-6*-3’UTR (sense 5’-AAGACCCCACACCCCTCCTT-3’, antisense 5’-CAATAGCCAAAATAGTTATT-3’, all 0.6 µM, Biologio, Leiden, The Netherlands) were used to isolate the 3’-UTR fragments from a cDNA pool of various human tissues.

COS7 cells were transfected with 100 ng UTR reporter plasmid and 50 pmol microRNA mimics as detailed above. 48 h post-transfection, luciferase activity was assayed using the DualGlo Luciferase assay system (Promega, Madison, WI) and recorded for 500 ms on a Luminoskan ASCENT (Thermo Scientific, Waltham, MA) according to manufacturer’s instructions. Relative luciferase activity was calculated by dividing the luminescence from *Renilla* luciferase activity by the luminescence from firefly luciferase activity and normalized to control samples.

### chromatin-immunoprecipitation (ChIP) and assessment of histone modifications

Cells were harvested using accutase, pelleted and the chromatin crosslinked using 1% formaldehyde (37% F1268 Sigma-Aldrich) for 8 min. Crosslinking activity was quenched using 125 mM glycine (104201 Merck). Cell pellets were lysed on ice with SDS lysis buffer (1% SDS, 50 mM Tris HCl pH 8.0, 10 mM EDTA) supplemented with freshly added 100 mM protease inhibitor cocktail (Sigma Aldrich P8340) for 15 min. The chromatin was fragmented by Biorupter (Diagenode, Seraing, Belgium) with five cycles of (30’ ON/OFF). The sonicated sample was centrifuged and chromatin containing supernatant was kept for further analysis. The chromatin was diluted 10 times with RIPA buffer (0.1% SDS, 0.1% Sodium deoxycholate, 1% Triton-X100, 1 mM EDTA, 10 mM Tris–HCl pH 7.5, 140 mM NaCl, 0.5 mM EGTA) supplemented with 100 mM protease inhibitor cocktail. Immunoprecipitation was performed by 4 µg H3K27Me3 antibody (Merk Millipore 07-449) or IgG control (Abcam ab46540) added to the 40uL Dynabeads Protein-A (Life technologies, 10002D) coated tubes. Subsequently, the chromatin of 0.8 × 10^6^ cells was added to antibody bound beads and incubated overnight at 4 C while rotating. The beads were washed 3 times with ice cold PBS and the remaining complexes were eluted with 100 nM NaHCO_3_ and 1% SDS in PBS. 5 M NaCl and RNAse (Roche #11119915001) were added to the eluted samples and incubated at 62 C to reversing the crosslink for 4 h. 2 µL Proteinase K (Roche #03115828001) was added and incubated at 62 C for 1 h to liberate the DNA from the histones. DNA fragments were purified using a QIAquick PCR purification kit (Qiagen) according to manufacturers’ instructions. Precipitated DNA was analyzed by qPCR using seven sets of primers for each promoter area. (Table 3; all 0.6 µM Biolegio) Enrichment of promoter sequences in the precipitate were calculated relative to the percentage of input.

**Table 3:** Primers used for CHIP-qPCR assays.

Genomic region	Chr	Sense sequence	Antisense sequence
***miR-200b/a/429***			
−2.5 kb	1	GGAGGAGCTGGTGTGTTCTC	CAAAGCCGCCATTTCACC
−2.0 kb	1	GCGGTGATGATTAACCCAAC	GTGGCCACAGGTCAAGAAAT
−1.5 kb	1	GGTGAGAACGCAATGACTGA	CTCCCACTGCCAGGTTCA
−1.0 kb	1	TTGGAGGAGGAGACTGGAAC	AGTTTTCTGGCACCTTCCAC
−0.5 kb	1	GACCAGCAGACACACAAACC	GACCCCTCTCCCATGCTG
TSS	1	TACTGAGCTTCCCAGCGAGT	AATGCTGCCCAGTAAGATGG
+ 0.5 kb	1	GAGCAGGACCCAACAGAGG	CAGGAGGGAAGATGGCTGT
***miR-200c/141***			
−2.5 kb	12	CACCTCCAGGTTCCAGCTAC	AAATGCTTCCACAGGGTCAG
−2.0 kb	12	ACAGGGTGGTTGTGAAAAGC	CGCCACGGTAAAATGAGAAT
−1.5 kb	12	CATGTCAGGAGTGGGGTTTC	CTTTGGGACCTGTGCTGTCT
−1.0 kb	12	GACTCCACTGAGGGCTGTG	TGAAGTTACCCACCGCTACC
−0.5 kb	12	GCCTAGAGGAGTGGCCAAG	GGTGTGTCCTCCTGCCATAG
TSS	12	AGGGCTCACCAGGAAGTGT	AGGATCCCTGCGGAAAAG
+ 0.5 kb	12	CCCTGTAGCAACTGGTGAGC	GGGAGCCATCTTTACCAGAC
***miR-101-1***			
−2.5 kb	1	CCACAGGCCTGGTTGTAGAT	AATCATTGGCCTTGGTGAAG
−2.0 kb	1	TGGGTAGAGCAGAGGGAAGA	ATCCTTCATTGTGCCAGTCC
−1.5 kb	1	TGACCGCAGACTGAGACTCTT	GCCAGGGAGAGAAAAACCAT
−1.0 kb	1	TGGAGGTTGAAGATGTGTGC	CTGGTCTTGACCTCCTGAGC
−0.5 kb	1	TGACAGCAGCAGCAATAACA	CCAGCTTACTGAAGTGAAGAAAGA
TSS	1	TTCTTCCTGGGTACGGTGAG	CCGACACAGTGACTGACAGG
+ 0.5 kb	1	ACTGACTGTGCCTCCCTGAC	TGAGCACTTTGAAGACAGGA
***DUSP-1 (MKP-1)***			
−2.5 kb	5	CCTCACCCCTGCTCTTTATG	ATGGCTTGCAGTGACCTTCT
−2.0 kb	5	CAGCAAGGGAGGAGAGAGAA	GCCTGGTGACAGAGCAAGAC
−1.5 kb	5	GCCTCGCTTAGCTTGTGTGT	CACTCCATGCCCTGAACTTT
−1.0 kb	5	GCAGTGGATTCCAGGGTTT	GAAAGGGATGGAGAAGCTCA
−0.5 kb	5	GCTTCCTGTGCTTTTGCATAC	CCCCAGTAGTGTGGTTCTGG
TSS	5	CGCTTTTGGACTGAGAGAGG	CTCGCTGCGAAGGACATT
+ 0.5 kb	5	AGGGCGTACCTTTGAGGAAG	GTGGTGTTGCTGGACGAG
***DUSP-6 (MKP-3)***			
−2.5 kb	12	AGGCCTAGGTTGCCAATTTT	AAAATGGTGCGGAGAGGAG
−2.0 kb	12	ATTGGAAGCCGGATGGAG	GCAGGCTTCGGCACTTTTAT
−1.5 kb	12	AATGATTTCTGGGCAAGGAG	GGTCTTGCGGGAGGACTT
−1.0 kb	12	GACCCAAGTTCGCCTTAACC	ACACAGCCTCGGCTAAAAGA
−0.5 kb	12	AGGCAGCTCCTCAATGGATA	TCATCAACACAACCTGTTCCA
TSS	12	GTCTTGCTGATCGCCATTTC	AGCTCGACCCCCATGATAG
+ 0.5 kb	12	GGAAGCGAGTGGATTCTGAG	CGCGTGGATTGAAAATACCT

### Angiogenic sprouting assay

10µL Matrigel (BD Corning, 356230) was added into the bottom compartment of µ slide Angiogenesis (81501, Ibidi GmbH, Martinsried, Germany) and incubated at 37 C, 5% CO_2_ for 1 h. Cells were diluted to 2 × 10^6^ cells/ml. 50 µl cell suspension was added on top compartment. After 6 h incubation at 37 C, 5% CO_2,_ light microscopy images were obtained, and complete octamer niches were counted by eye.

### Collagen contraction assay

Cells were dissociated using trypsin–EDTA, pelleted and suspended at a concentration of 22.5 × 10^6^ cells/ml ECM. 45 µL cell suspension was added to a collagen solution (3.3 mg/ml rat tail collagen type I (#354236, BD, San Jose, CA), 100 mM Na_2_HPO_4_ and 5 mg/ml NaHCO_3_) of neutral pH. The cell/collagen mixture was immediately aliquoted into 50 µl droplets and allowed to polymerize at 37 C, 5% CO_2_ for 30 min. Polymerized gels were released and 1 mL of ECM was added. At time points t = 0 h and t = 24 h, gels were visualized using a regular flatbed scanner and the gel surface area quantified using with ImageJ (NIH). Gel contraction was calculated as the relative reduction in gel surface area at 24 h.

### Permeability assay

Cells (5 × 10^4^/cm^2^) were cultured on polycarbonate cell culture inserts strips (pore size 0.4 µm, porosity, 0.9 × 10^8^/cm^2^ Fisher Scientific, #15639536) coated with 0.1% gelatin for 72 h to establish a monolayer. Monolayer permeability was assessed by the addition of 5 µg/mL FITC dextran (Sigma) in upper compartment. Fluorescence was measured in the bottom compartment on fluorescence reader at Ex485/Em519 30 min after the addition of dextran. Relative permeability levels were calculated using the fluorescence signal of a naked strip (100% permeability) or the fluorescence signal from the culture medium (0% permeable). Permeability was calculated by following formula: Permeability = (Em_519_(sample) − Em_519_(ECM))/Em_519_(Empty well) × 100.

### Data representation of statistical analyses

Data are expressed as mean ± s.e.m. from at least three independent experiments. Where the mean of two groups were compared, p-values were calculated using student t-tests. Otherwise, p-values were calculated using the one-way analysis of variance (ANOVA) followed by Bonferroni's post-hoc comparisons tests using GraphPad Prism 9 (GraphPad Software, La Jolla, CA, USA). P < 0.05 was considered statistically significant.

## Supplementary Information

Supplementary Figures.

## Data Availability

All data generated or analyzed during this study are included in this published article. Materials, data, and associated protocols are available from the corresponding author on reasonable request without preconditions.
