# Supplementary material for: Reciprocal regulation of endothelial–mesenchymal transition by MAPK7 and EZH2 in intimal hyperplasia and coronary artery disease
Source: Sci Rep. 2021 Sep 7;11:17764. doi: 10.1038/s41598-021-97127-4 (PMC8423795; doi:10.1038/s41598-021-97127-4)
Supplement: Supplementary file 1 — Supplementary Figures. [file 41598_2021_97127_MOESM1_ESM.pdf]

Data Supplement – Vanchin *et al.* Reciprocal regulation of Endothelial-Mesenchymal Transition by MAPK7 and EZH2 in Intimal Hyperplasia and Coronary Artery Disease.

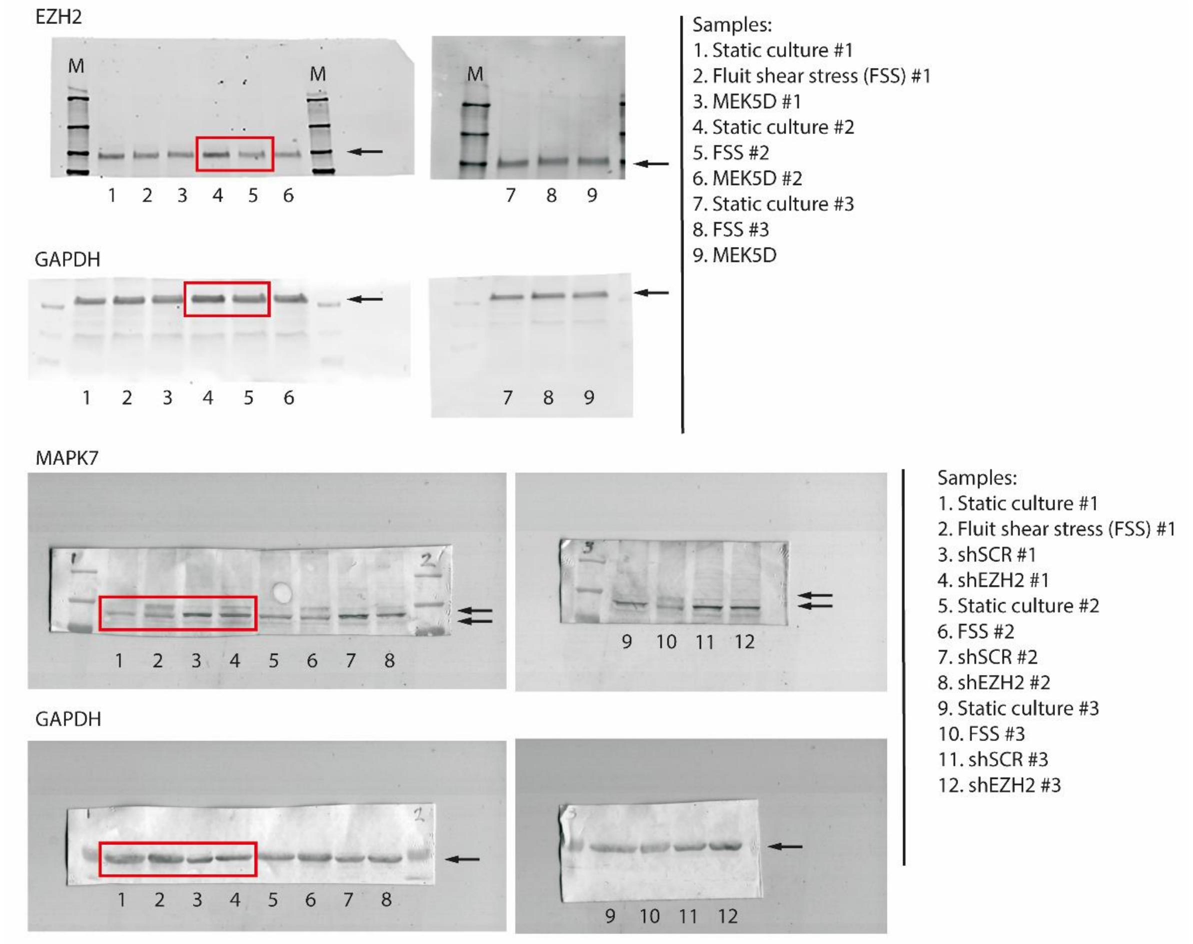

Supplemental figure 1. Annotated immunoblots of manuscript figure 2. Annotated immunoblots for EZH2, MAPK7 and GAPDH for sample replicates summarized in manuscript figure 2. Numbers specify the individual samples, arrows indicate the appropriate band sizes and red boxes annotate the bands used in the manuscript.

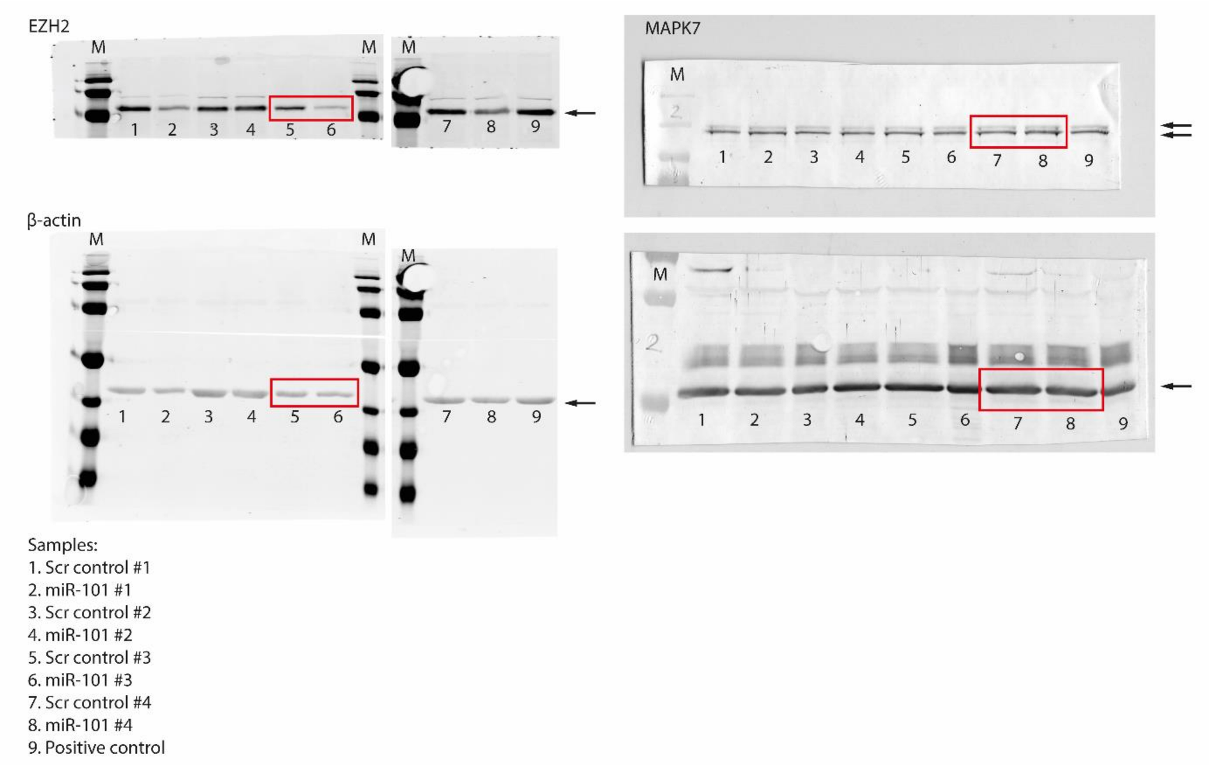

Supplemental figure 2. Annotated immunoblots of manuscript figure 3. Annotated immunoblots for EZH2, MAPK7 and β-actin for sample replicates summarized in manuscript figure 3. Numbers specify the individual samples, arrows indicate the appropriate band sizes and red boxes annotate the bands used in the manuscript.

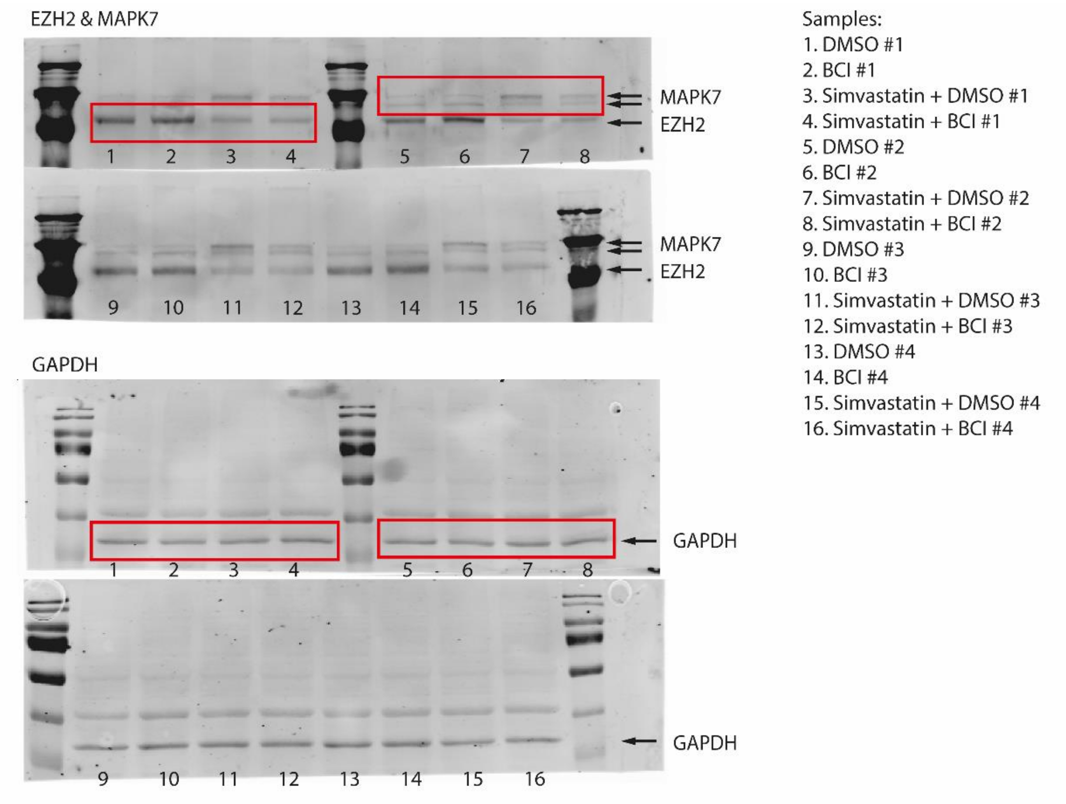

Supplemental figure 3. Annotated immunoblots of manuscript figure 6. Annotated immunoblots for EZH2, MAPK7 and GAPDH for sample replicated summarized in manuscript figure 6. Numbers specify the individual samples, arrows indicate the appropriate band sizes and red boxes annotate the bands used in the manuscript.

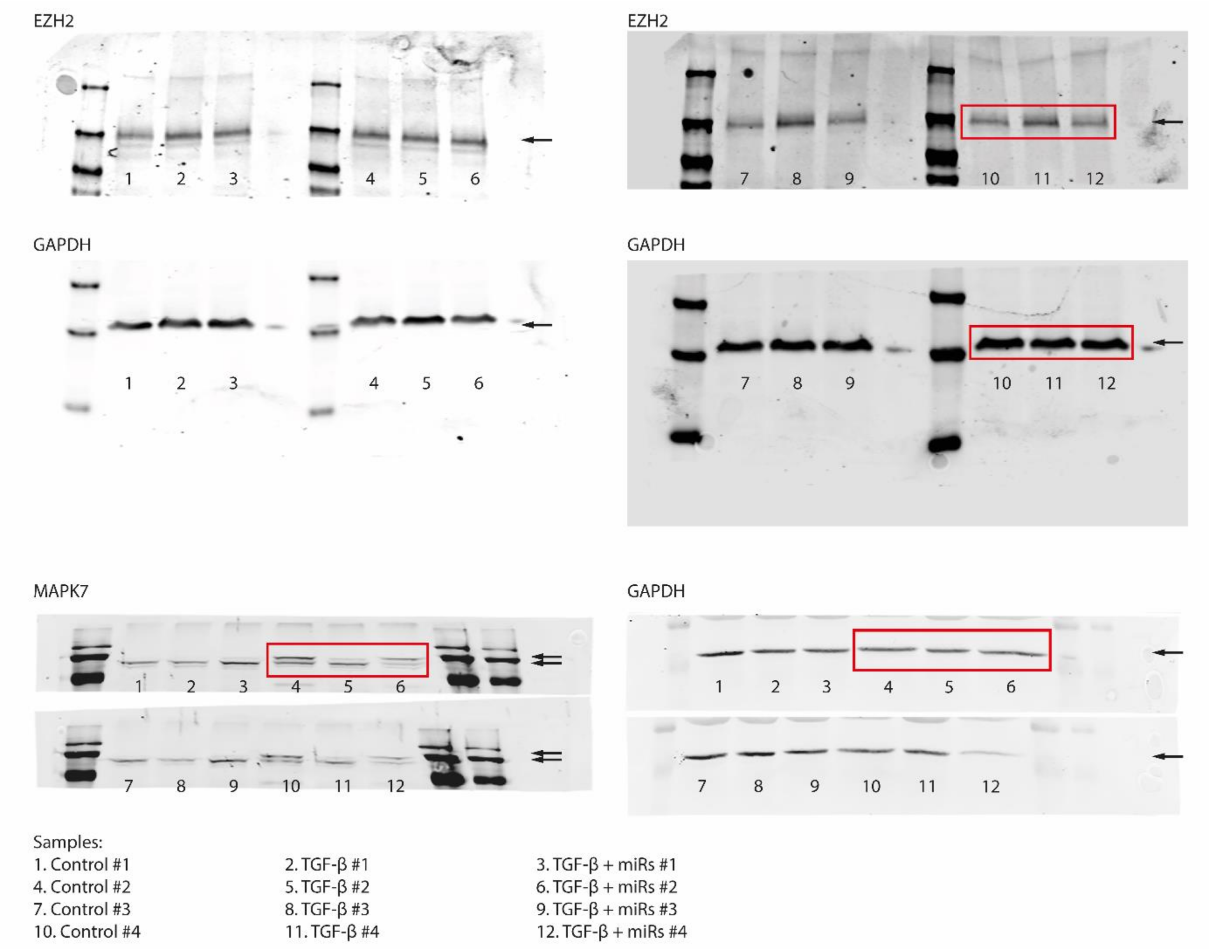

Supplemental figure 4. Annotated immunoblots of manuscript figure 7. Annotated immunoblots for EZH2, MAPK7 and GAPDH for sample replicated summarized in manuscript figure 7. Numbers specify the individual samples, arrows indicate the appropriate band sizes and red boxes annotate the bands used in the manuscript.
